# Is High Whitefly Abundance on Cassava in Sub-Saharan Africa Driven by Biological Traits of a Specific, Cryptic *Bemisia tabaci* Species?

**DOI:** 10.3390/insects12030260

**Published:** 2021-03-20

**Authors:** Habibu Mugerwa, Peter Sseruwagi, John Colvin, Susan Seal

**Affiliations:** 1 Natural Resources Institute, University of Greenwich, Central Avenue, Chatham Maritime, Kent ME4 4TB, UK; j.colvin@gre.ac.uk; 2 Department of Entomology, University of Georgia, 1109 Experiment Street, Griffin, GA 30223, USA; 3 Biotechnology Department, Mikocheni Agricultural Research Institute, P.O. Box 6226 Dar es Salaam, Tanzania; psseruwagi@yahoo.co.uk

**Keywords:** whitefly, biological traits, superabundance, management implications

## Abstract

**Simple Summary:**

Whiteflies are pests and vectors of plant viruses which cause devastating yield losses globally to numerous food, cash and ornamental crops. The main whitefly (*Bemisia tabaci*) populations that infest cassava, a food security crop for sub-Saharan Africa (SSA), can reach highly abundant populations and are SSA1 and SSA2. SSA1 has been divided into separate species/subgroups (SG1/SG2 and SG3) and these can differ in their abundance and prevalence in the field in east Africa. In this study, biological traits of 12 whitefly populations collected from Uganda and Tanzania were investigated to determine whether their genotypes alone are significant drivers of the observed field abundance. Development of *B. tabaci* populations (SSA1 subgroups SG1/SG2 and SSA2) under insectary conditions were similar, hence not supporting previous suggestions of SSA1-SG1 being predominant in eastern Africa, due to distinct biological traits. SSA1-SG3 development time was the shortest, whereas SSA1-SG2 generated the greatest number of emerged adults. These results assist understanding of factors contributing to the outbreaking phenomenon of whitefly populations that drive cassava-virus pandemics in eastern Africa.

**Abstract:**

In East Africa, the prevalent *Bemisia tabaci* whiteflies on the food security crop cassava are classified as sub-Saharan Africa (SSA) species. Economically damaging cassava whitefly populations were associated with the SSA2 species in the 1990s, but more recently, it has been to SSA1 species. To investigate whether biological traits (number of first instar nymphs, emerged adults, proportion of females in progeny and development time) of the cassava whitefly species are significant drivers of the observed field abundance, our study determined the development of SSA1 sub-group (SG) 1 (5 populations), SG2 (5 populations), SG3 (1 population) and SSA2 (1 population) on cassava and eggplant under laboratory conditions. SSA1-(SG1-SG2) and SSA2 populations’ development traits were similar. Regardless of the host plant, SSA1-SG2 populations had the highest number of first instar nymphs (60.6 ± 3.4) and emerged adults (50.9 ± 3.6), followed by SSA1-SG1 (55.5 ± 3.2 and 44.6 ± 3.3), SSA2 (45.8 ± 5.7 and 32.6 ± 5.1) and the lowest were SSA1-SG3 (34.2 ± 6.1 and 32.0 ± 7.1) populations. SSA1-SG3 population had the shortest egg–adult emergence development time (26.7 days), followed by SSA1-SG1 (29.1 days), SSA1-SG2 (29.6 days) and SSA2 (32.2 days). Regardless of the whitefly population, development time was significantly shorter on eggplant (25.1 ± 0.9 days) than cassava (34.6 ± 1.0 days). These results support that SSA1-(SG1-SG2) and SSA2 *B. tabaci* can become highly abundant on cassava, with their species classification alone not correlating with observed abundance and prevalence.

## 1. Introduction

*Bemisia tabaci* (Hemiptera: Aleyrodidae) species of whitefly are phloem-feeding insects that cause widespread damage to crops and weeds, including cassava [1,2,3], a staple food crop for millions of people in Africa [4,5]. Damage is caused primarily by members of the *B. tabaci* cryptic species complex that collectively vector more than 400 plant viruses globally [6]. The *B. tabaci* species that colonize cassava vector the viruses that cause the devastating cassava mosaic disease (CMD) and cassava brown streak disease (CBSD) [7,8]. Estimated economic losses attributed to CMD and CBSD in sub-Saharan Africa (SSA) are US$1.9–2.7 billion and US$0.1 billion annually, respectively [9,10].

In the 1990s, the spread of viruses that caused the severe CMD pandemic in Uganda was associated with exceptionally high whitefly populations [11,12,13]. Areas severely affected by the CMD pandemic had high whitefly populations, whose identity was determined to be Uganda 2 [11,14], currently referred to as Sub-Saharan Africa 2 (SSA2) [15]. Areas not yet affected by CMD termed ‘non-pandemic zones’ had low whitefly populations, whose identity was determined to be Uganda 1 [11,14], currently referred to as SSA1 [15]. Subsequent studies, however, found no significant fecundity differences among CMD pandemic and non-pandemic populations [16]. Further, just a few years later, the presence of both SSA1 and SSA2 species on cassava in the post-pandemic areas of Uganda were reported [17]. Contrary to the earlier study carried out by Legg et al. [11], SSA1 occurred more frequently (83%) than SSA2 (17%) species and no definite association could be established between these species and the cassava mosaic begomoviruses (CMBs) present at the time [17]. In another comprehensive study carried out on whitefly specimens collected from cassava in East and Central Africa between 1997 and 2010, SSA2 *B. tabaci* were the prevalent species on cassava between 1997 to 1999, but this shifted to SSA1 from 2000 to 2010 [4]. In the Legg et al. [4] study, a terminology ‘subgroup’ (SG) was introduced to refer to genotypes within SSA1 species that diverged by ~1.9–2.0% nt in their partial *mtCO1* sequences used for barcoding.

Three of the Legg et al. [4] subgroups (SG1 to SG3) of SSA1 *B. tabaci* have been shown to be the predominant whiteflies occurring on cassava in East Africa in the past decade [4,18,19]. While three subgroups (SG1, SG2 and SG3) of *mtCO1* sequences occur within the SSA1 species, different studies have shown that this taxonomic marker widely used by the whitefly research community is in some cases not accurate for species delimitation [20,21]. The use of whole genome SNPs markers and reciprocal crossing assays, as well as microsatellite analyses, have confirmed that SSA1-SG1 and SSA1-SG2 populations belong to the same species, which is a distinct species to SSA1-SG3 [19,21].

In terms of field abundance, the cassava whitefly species and subgroups thereof have differed quite markedly over the past two decades in various studies. SSA1-SG1 and SSA1-SG2 populations occurred at 25% and 11% frequency respectively, between 1997 to 1999 on cassava, while SSA2 occurred at 64% [4]. From 2001 to 2010, SSA1-SG1 frequency increased to >76%, while that of SSA1-SG2 and SSA2 species were reported at 12% and 10% respectively, in East and Central Africa [4]. Whitefly samples collected on cassava in central Uganda in 2012 identified SSA1-SG1 and SSA1-SG2 populations occurring at 69% and 31% frequency, whilst 35 samples collected from southern Tanzania in 2012 and 2014 were all SSA1-SG3 [18]. Further, the SSA1 species was among the 21 whitefly species identified in a country-wide survey on 870 whitefly adults collected from >60 plant species in 2013 in Uganda, with a composition of SG1 = 16%, SG2 = 4% and SG3 = 3% [22]. Contrasting results were obtained by a separate study that found SSA1-SG1 and SSA1-SG2 occurred at almost equal frequencies (51% and 49%, respectively), while SSA2 whiteflies were not detected at all in central Uganda in 2017 [19]. Recently, a study by MacFadyen et al. [23] on whitefly samples (nymphs and adult flies) collected from mainly cassava plants across Malawi, Tanzania and Uganda, in 2015 and 2016, revealed SSA1 as the prevalent whitefly species on cassava.

Contrasting results from field surveys are not surprising, with a range of factors including environment and climate influencing the abundance, distribution, life history and fitness of whitefly populations in a landscape. For example, rapid leaf growth caused by high temperatures and rainfall boost whitefly populations [5,24,25,26]. Young (1–4 months of age) cassava plants harbor more whitefly than older (>5 months) cassava plants [27]. Improved cassava varieties generated through classical plant breeding also tend to support higher whitefly populations than local landraces [26,28]. The presence of natural enemies negatively impacts whitefly populations [23,29]. Exposure to high temperatures (>40 °C) negatively impacts whitefly fitness and survival [30,31]. Further, the ratio of male to female *B. tabaci* varies greatly under field conditions and it is affected by host plant, temperature and the time of the year [32,33]. To gain an improved understanding of how biological traits of whitefly populations affect their field abundance, this needs to be studied under standardized laboratory conditions.

Laboratory studies comparing high *B. tabaci* cassava whitefly populations from CMD pandemic zones to less abundant cassava whitefly populations from non-pandemic zones of Uganda in the late 1990s have been undertaken previously [16]. Using *B. tabaci* colonies set up from 100–150 whiteflies collected from cassava fields located in CMD pandemic and non-pandemic zones, there were no significant (*p* > 0.05) fecundity differences among CMD pandemic and non-pandemic populations. Our study built upon these studies but used *B. tabaci* colonies set up from individual male and female whiteflies of known *mtCO1* identity, to overcome the possibility of a mixture of genotypes in populations from multiple individuals. Mixed populations of whitefly species are commonly detected from cassava [11,12,13], and hence, this modification allowed us to investigate whether, or not, field abundance is dependent on differences in the development of a given whitefly *mtCO1* genotype.

A screen-house study conducted by Mugerwa et al. [34] demonstrated that an SSA1-SG1 population (associated with high abundance in the Lake Victoria basin of Tanzania) had a significantly higher fecundity than an SSA1-SG3 population, a *B. tabaci* species associated with low whitefly populations in coastal areas of Tanzania. It is, however, unknown whether SSA1-SG2 and SSA2 *B. tabaci* species possess similar or inferior development traits in relation to SSA1-SG1 and SSA1-SG3 whiteflies. Although some studies [4,18,19] suggest that SSA1-SG2 can be highly fecund, comparative studies under standardized conditions from colonies set up from single females are required to shed light on field observations.

In this study, SSA1-SG1 and SSA1-SG2 (currently associated with high populations in East Africa) [4,18,19,23], SSA2 (associated with high populations in the 1990s in Uganda) [4,11] and SSA1-SG3 *B. tabaci* (currently associated with low populations in the coast zone, Tanzania) [18,34] were studied to determine development differences among them. To test the hypothesis that variation in field abundance can be associated with specific *B. tabaci* genotypes (SSA1-SG1/SG2, SSA1-SG3, SSA2), we measured their development in a controlled environment insectary. Understanding the factors behind the generation of high whitefly populations is important because their occurrence leads to increased crop physical damage and disease spread, which threaten food security and income generation in sub-Saharan Africa.

## 2. Materials and Methods

### 2.1. Generation of Host Plants

Soil mixture made up of multipurpose Jiffy substrates (Jiffy products, Berkshire, UK) and John Innes No. 2 compost (John Innes, Norwich, UK) in a 50:50 ratio was sterilized by autoclaving (121 °C, 15 min). The soil mixture was put in plant pots of 4 cm in diameter. Tissue-cultured cassava plantlets of cultivar Colombian were carefully removed from growth media and gently washed in water at 25 °C to remove all growth media. Older leaves of the plantlets were trimmed off, leaving two young leaves. The plantlets were transferred to soil in pots. The plants were put in trays that were covered with transparent lids with vents to minimize water loss from the plants. The vents were opened 14 days after transferring the plantlets and the plants were ready for use after 30 days of establishment.

Eggplant seeds, variety Black Beauty (Kings Seeds, Coggse-shall, UK), were planted in the aforementioned soil mixture. After 20 days at 25 °C under controlled environment of 12 h light:12 h darkness, eggplant plants had 3‒4 fully formed leaves and were ready for use.

### 2.2. Establishment of B. tabaci colonies

Whitefly colonies were set up as described by Mugerwa et al. [21]. In brief, adult *B. tabaci* were collected from cassava fields in six different locations within the Lake Victoria Crescent in Uganda and a single location in Dar es Salaam in the Coast zone in Tanzania (Table 1). SSA1-SG1 and SSA1-SG2 were collected from five locations within the same agro-ecological zone (Lake Victoria Crescent) in order to examine why different levels of field abundances of the same genotype occur in a given agro-ecological zone. Adult *B. tabaci* (n = 100–200) were collected from each location using an aspirator and transferred to “Lock & Lock” (LL) containers [35] having a cassava shoot with tender leaves. After transport to the quarantine insectaries at the Natural Resources Institute (NRI, UK), the surviving *B. tabaci* were transferred to LL containers with 20-day-old eggplant plants to initiate field-collected colonies of unknown and mixed identities. Live whiteflies were collected and put individually into a 5 × 0.5 cm glass tube covered with cotton wool to prevent escape. The sex of the collected whiteflies was determined by looking at the shape of the whitefly abdomen using a Wild M8 stereomicroscrope (Wild Heerbrugg, Switzerland). Ten pairs (female and male) of whiteflies were collected from each of the seven field-collected colonies to set up isofemale lines (10 × 7 = 70 isofemale lines). Upon establishment, their identity was determined using the partial *mtCO1* marker as described in the following paragraphs. Based on the reproductive vigor of the established isofemale line and its *mtCO1* identity, 12 isofemale lines representing SSA1-SG1, SSA1-SG2, SSA1-SG3 and SSA2 species were selected for fecundity studies. The 12 isofemale lines included five *B. tabaci* lines for both SSA1-SG1 and SSA1-SG2, representing five collection sites in Uganda, which were Kayingo (KAY), Kisubi (KIS), Kyabakadde (KYA), Nakisunga (NAK) and Namulonge (NAM), and a single isofemale line for each of SSA1-SG3 and SSA2 *B. tabaci* collected from Dar es Salaam (DAR), Tanzania and Kiboga (KIB), Uganda, respectively. The 12 selected isofemale lines were transferred to BugDorm insect-rearing rectangular cages (47.5 × 47.5 × 93 cm, MegaView Science Co., Ltd., Taiwan) and maintained at 28 ± 2 °C, 60% relative humidity (r.h.) and 12:12 L:D.

The SSA1-SG1, SSA1-SG2 and SSA2 *B. tabaci* were collected from locations belonging to the Lake Victoria Crescent agro-ecological zone, while the SSA1-SG3 was collected from a location belonging to the Coast agro-ecological zone. The Lake Victoria Crescent has bimodal rainfall averaging 1000 mm annually, temperatures ranging from 25 to 31 °C, an average altitude of 1170 m and the vegetation is savannah grassland with moderate biomass [36,37]. The Coast zone has a bimodal rainfall ranging from 750 to 1200 mm annually, temperature ranging from 22 to 30 °C, altitude of under 300 m and a savannah grassland (https://web.archive.org/web/20170207141047/http://kilimo.go.tz:80/agricultural%20maps/Tanzania%20Soil%20Maps/Soil%20maps.htm) (accessed on 1 February 2021).

### 2.3. Extraction of DNA from an Individual Whitefly

Genomic DNA was extracted from individual whiteflies by crushing the adult fly in 50 µL of 10% (*w*/*v*) Chelex^®^ 100 sodium form solution (Sigma Aldrich, St. Louis, MO, USA) in a sterile 1.5 mL Eppendorf tube using a sterile plastic pestle according to the procedure of Walsh et al. [38]. The 1.5 mL Eppendorf tube with the extract was incubated for 20 min at 56 °C, followed by 5 min at 100 °C. Subsequently, the tubes were centrifuged (15,900× *g*, 5 min) and placed on ice prior to being used as DNA template for polymerase chain reaction (PCR) amplifications.

### 2.4. Polymerase Chain Reaction (PCR), Gel Electrophoresis and Sequencing

Amplification of a partial fragment (867 bp) of the *mtCO1* gene was performed using the forward primer 2195Bt (5′-TGRTTTTTTGGTCATCCRGAAGT-3′) in combination with a reverse primer C012/Bt-sh2 (5′-TTTACTGCACTTTCTGCC-3′) [1]. PCR reaction mixtures (20 µL) were set up containing 10 µL of 2 × reSource™Taq Mix (reSource Taq DNA polymerase, 6 mM MgCl_2_, 2 mM dNTPs, enhancers and stabilizers) (Source BioScience, Nottingham, UK), 1 µL of each 10 µM primer, 6 µL molecular grade water and 2 µL DNA template. A negative control (molecular biology grade water used in the place of DNA template) was included in every PCR run. PCR cycling was performed in a 2720 Thermal cycler (Applied Biosystems, Foster City, CA, USA) programmed as follows: Initial denaturation at 94 °C/2 min, 35 cycles of 94 °C/30 s, annealing at 52 °C/30 s and extension at 72 °C/1 min, with a final 10 min elongation cycle at 72 °C. PCR products were visualized through stained agarose gel after electrophoresis under ultra violet (UV) light. Amplified PCR products of expected size were purified using a resource PCR purification kit as per manufacturer’s instructions (Source BioScience, UK). Purified PCR products were sent for Sanger sequencing at Source Bioscience (Nottingham, UK).

### 2.5. Analysis of Sequence Data

Obtained sequences were edited manually using the MEGA version 6 (v6) software program [39]. Edited sequences were used for similarity searches in the National Centre for Biotechnology Information (NCBI) GenBank databases (www.ncbi.nlm.nih.gov) (accessed on 1 February 2021). Edited sequences were also aligned together with reference whitefly sequences obtained from GenBank using Clustal W [40] available in MEGA v6 [39]. Aligned sequences were trimmed to 657 bp and the best-fit model to analyze the data was determined in MEGA v6. The phylogeny tree was generated in MEGA v6 using the maximum likelihood (ML) statistical method with rates among sites variation corresponding with gamma distribution and a general-time-reversible substitution model. Nucleotide divergence among sequences was performed through visual inspection of the 794 bp sequences within and between sequence groups.

### 2.6. Development Experiment Procedure

For each assay, virgin females and males of the same location population were paired together. Each experiment was replicated five times. In some cases, one or two replicates out of the five failed and hence reliable data was only obtained for 3–4 replicates for such treatments. The total number of treatments was 12, representing the 12 populations established in the study. This experiment was carried out on 20-day-old eggplant plants and 30-day-old cassava plants trimmed to a single leaf before transferring to a LL container.

A single virgin female (♀) and three virgin male (♂) adults in glass tubes were introduced to the eggplant or cassava plant in an LL container in the insectary. At two-day intervals, plants were inspected, and in cases where a male had died, it was replaced with a live one. After seven days of feeding and female ovipositing, all the whiteflies were removed from the eggplant or cassava plant in the LL container and stored in 90% ethanol. Eggs laid were left to develop to fourth instar nymphs. The plants were inspected for emerged adults every day from day 20 to day 65. The numbers of first instar nymphs, emerged male and female adult offspring were recorded, and the adults were stored in 90% ethanol. Due to a wide adult eclosion time, average development time for each replicate was calculated as sum product (emergence days, number of emerged adults for a given day)/sum (total number of emerged adults). The development experiment was carried out under climatic conditions stated in Section 2.2.

### 2.7. Data Analysis for the Development Experiment

The development experiment (Raw data S1 and S2) was analyzed in three ways: (i) the average number of first instar nymphs and emerged progeny produced by whiteflies from a given population and location, (ii) the proportion of females in emerged progeny and (iii) the development time (egg to adult) of progeny for a given population and location. Only the proportion of females in emerged adults was considered and analyzed in this study since the number of female offspring determine the population size of the next generation. In the comparison of SSA1-SG1, SSA1-SG2, SSA1-SG3 and SSA2 populations on eggplant and cassava, all SSA1-SG1 and SSA1-SG2 data (number of first instar nymphs and emerged progeny, proportion of females in emerged progeny and development time) generated for the five locations were pooled together. Statistical analyses were performed using R [41] (Scripts S1 and S2). The first instar nymphs and progeny counts were analyzed using a negative binomial generalized linear model with log link in the MASS package [42]. The proportion of females in emerged progeny were analyzed using a quasibinomial generalized linear model with binomial errors and logit transformation in the MASS package. Development time was analyzed using analysis of variance available in R. A Tukey HSD test was used for multiple comparisons using the multcomp package [43] to determine significant differences between development time, nymph and progeny counts and proportion of females in the emerged progeny. Multiple comparisons were displayed by compact letter display (cld) using the cld function.

## 3. Results

### 3.1. Molecular Identity of Whitefly Colonies

**Figure 1:**
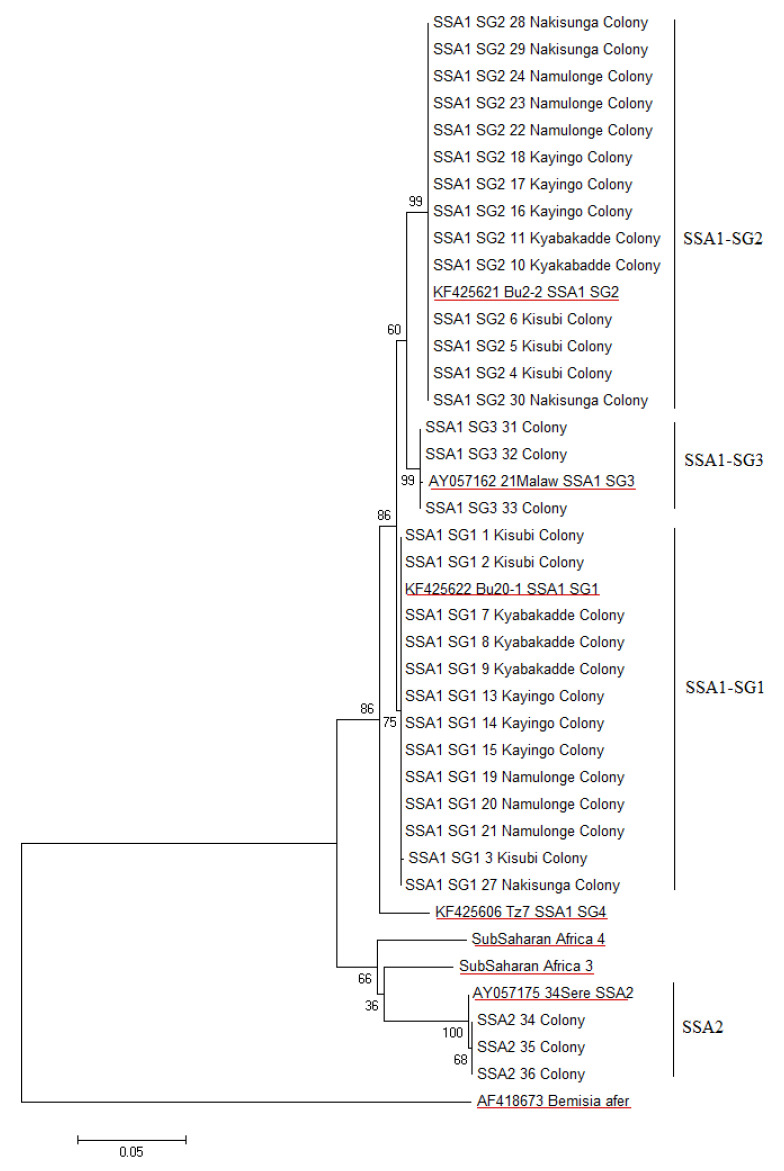
Maximum Parsimony phylogeny reconstruction based on partial *mtCO1* sequences (657 bp) of individual *Bemisia tabaci* collected from colonies established at the Natural Resources Institute (NRI). The location of collection of individual *B. tabaci* SSA1-SG1 and SSA1-SG2 in Uganda is denoted in the sequence name. Individual *B. tabaci* 31–33 were collected from the SSA1-SG3 colony established from Dar es Salaam (Tanzania). Individual *B. tabaci* 34–36 were collected from SSA2 colony established from Kiboga (Uganda). Underlined are reference sequences from Legg et al. [4]. *B. afer* was used as an outgroup.

A phylogenetic analysis of the *mtCO1* sequences of the *B. tabaci* whiteflies obtained from the 12 established colonies grouped into two low-level genetic groups, namely SSA1 and SSA2 (Figure 1), as described by Dinsdale et al. [15]. Within the SSA1 *B. tabaci*, three populations: SSA1-SG1, SSA1-SG2 and SSA1-SG3, were identified in line with the naming system used by Legg et al. [4]. Partial *mtCO1* sequences of SSA1-SG1 *B. tabaci* diverged from SSA1-SG2 *B. tabaci* by 13/794 nt. A partial *mtCO1* sequence of a whitefly collected from the SSA1-SG3 *B. tabaci* colony established from Dar es Salaam, Tanzania, diverged from SSA1-SG1 and SSA1-SG2 *B. tabaci* partial *mtCO1* sequences by 10/794 nt and 13/794, respectively. Partial *mtCO1* sequences of individual SSA1-SG1 and SSA1-SG2 *B. tabaci* collected from the five whitefly colonies from Uganda were 100% (794/794 nt) identical within each subgroup. A partial *mtCO1* sequence of a whitefly collected from the SSA2 colony diverged from SSA1-SG1, SSA1-SG2 and SSA1-SG3 *B. tabaci* partial *mtCO1* sequences by 60/794, 64/794 and 60/794 nt, respectively.

### 3.2. Growth and Development of SSA1-SG1, SSA1-SG2, SSA1-SG3 and SSA2 B. tabaci

The effect of biotic factors (host plant and *B. tabaci*) on the growth and development of *B. tabaci* SSA1-SG1, SSA1-SG2, SSA1-SG3 and SSA2 populations were studied in the NRI insectary in 2016.

#### 3.2.1. First Instar Nymphs

Host plant effect did not significantly (*p* = 0.1884) affect the number of first instar nymphs, although there were more *B. tabaci* first instar nymphs developing on cassava (58.8 ± 3.2) than on eggplant (53.0 ± 2.8) (Table 2). However, there were significant differences (*p* = 0.0099) in the number of first instar nymphs produced by the different *B. tabaci* populations. SSA1-SG2 populations (60.6 ± 3.4) had the highest number of first instar nymphs, followed by SSA1-SG1 (55.1 ± 3.2) and SSA2 (45.8 ± 5.7) populations. The SSA1-SG3 population (34.2 ± 6.1) had the least number of first instar nymphs developing from eggs. There was no significant effect (*p* = 0.5662) in the *B. tabaci* population × host plant interaction on the number of first instar nymphs developing from eggs laid.

#### 3.2.2. Emerged Adults

Host plant did not have a significant (*p* = 0.05565) effect on the number of emerged adults in the experiment, despite slightly more *B. tabaci* adults emerging on eggplant (49.6 ± 3.3) than on cassava (41.2 ± 2.9) (Table 2). However, there were significant differences (*p* = 0.0274) in the number of emerged adults from the different *B. tabaci* populations. SSA1-SG2 populations (50.9 ± 3.6) had the highest number of emerged adults, followed by SSA1-SG1 (44.6 ± 3.3) and SSA2 (32.6 ± 5.1) populations. SSA1-SG3 population (32.0 ± 7.1) had the least number of emerged adults (Table 2). There was no significant effect (*p* = 0.0647) in the *B. tabaci* population × host plant interaction.

#### 3.2.3. Proportion of Females in Emerged Adults

Host plant did not have a significant (*p* = 0.4475) effect on the proportion of females that emerged, although there was a slightly higher proportion of females in emerged adults on cassava (0.44 ± 0.04) than on eggplant (0.41 ± 0.03). However, there were no significant (*p* = 0.4988) differences in the proportion of emerged female adults for the different *B. tabaci* populations, although SSA1-SG1 had a lower proportion of emerged female adults (0.39 ± 0.04) compared to SSA1-SG3 (0.51 ± 0.13), SSA2 (0.54 ± 0.09) and SSA1-SG2 (0.42 ± 0.03) populations (Table 2). There was no significant (*p* = 0.6709) effect in the proportion of emerged female adult *B. tabaci* population × host plant interaction.

#### 3.2.4. Development Time

Host plant had a significant (*p* < 0.0001) effect on *B. tabaci* developmental time, being shorter on eggplant (25.1 ± 0.9) than on cassava (34.6 ± 1.0) (Table 2). However, there were no significant differences (*p* = 0.6337) in the development times of the different *B. tabaci* populations. SSA1-SG3 (26.7 ± 3.7) had the shortest development time, followed by SSA1-SG1 (29.2 ± 1.2) and SSA1-SG2 (29.6 ± 1.2). SSA2 population (32.2 ± 2.6) had the longest development time (Table 2). There was no significant effect (*p* = 0.6672) in the *B. tabaci* population × host plant interaction on the development time.

### 3.3. Growth and Development of SSA1-SG1 and SSA1-SG2 B. tabaci

During sample collection, it was noticed that *B. tabaci* occurred in different abundances in the five locations sampled within the Lake Victoria crescent. Analysis of *mtCOI* showed the populations to comprise two genotypes: SSA1-SG1 and SSA1-SG2. To explore trait variation among these populations, we investigated their development under standardized insectary conditions.

#### 3.3.1. First Instar Nymphs

Area of collection (location) significantly (*p* = 0.0413) influenced the number of first instar *B. tabaci* nymphs. But the populations did not significantly (*p* = 0.2027) differ. Within SSA1-SG1, *B. tabaci* colonies established from Kisubi (65.3 ± 7.7) had the highest number of first instar nymphs, followed by Namulonge (62.1 ± 7.8), Nakisunga (56.9 ± 6.4) and Kyabakadde (47.6 ± 6.5). *B. tabaci* colonies established from Kayingo (43.7 ± 5.0) had the least number of first instar nymphs (Table 3). Within SSA1-SG2, *B. tabaci* colonies established from Nakisunga (72.4 ± 8.0) had the highest number of first instar nymphs, followed by Kayingo (69.9 ± 7.8), Kisubi (68.0 ± 8.5) and Kyabakadde (54.9 ± 6.2). *B. tabaci* colonies established from Namulonge (39.1 ± 4.5) had the least number of first instar nymphs, which were significantly (*p* < 0.05) different from Nakisunga, Kayingo and Kisubi. The location × *B. tabaci* population interaction effect was significant (*p* = 0.0017).

#### 3.3.2. Emerged Adults

Area of collection/location (*p* = 0.072) and *B. tabaci* population (*p* = 0.1383) had no significant influence on the number of emerged adults. Nonetheless, within SSA1-SG1, *B. tabaci* colonies established from Namulonge (53.2 ± 7.9) and Kisubi (51.7 ± 7.3) had the highest number of emerged adults, followed by Nakisunga (44.5 ± 6.0) and Kayingo (39.7 ± 5.4). *B. tabaci* colonies established from Kyabakadde (32.7 ± 5.4) had the least number of emerged adults (Table 3). For the SSA1-SG2, *B. tabaci* colonies established from Kayingo (63.6 ± 8.4) had the highest number of emerged adults, followed by Nakisunga (58.9 ± 7.8), Kisubi (57.8 ± 8.6), Kyabakadde (48.5 ± 6.5). *B. tabaci* colonies established from Namulonge (26.9 ± 3.8) had the least number of emerged adults which were significantly (*p* < 0.05) different from Nakisunga, Kisubi and Kyabakadde. The location × *B. tabaci* population interaction effect on emerged adults was significant (*p* = 0.0003).

#### 3.3.3. Proportion of Emerged Female Adults

Areas of collection/location significantly (*p* < 0.0001) influenced the proportion of emerged *B. tabaci* female adults. However, *B. tabaci* populations did not significantly (*p* = 0.6003) differ in the proportion of emerged female adults. Within SSA1-SG1, *B. tabaci* colonies established from Kayingo (0.68 ± 0.07) and Nakisunga (0.64 ± 0.07) had a significantly high proportion of emerged female adults compared to the other three locations (Table 3). Namulonge (0.27 ± 0.07), Kyabakadde (0.16 ± 0.07) and Kisubi *B. tabaci* colonies (0.16 ± 0.05) had the least proportion of emerged female adults. For SSA1-SG2, *B. tabaci* colonies established from Kayingo (0.50 ± 0.06) had the highest proportion of emerged female adults, followed by Nakisunga (0.43 ± 0.06) and Namulonge (0.43 ± 0.09). *B. tabaci* colonies established from Kisubi (0.38 ± 0.07) and Kyabakadde (0.35 ± 0.07) had the least proportion of emerged female adults. The location × *B. tabaci* population interaction effect on the proportion of emerged female adults was significant (*p* = 0.0011).

#### 3.3.4. Development Time

Areas of collection significantly (*p* = 0.0058) affected the development time of *B. tabaci* populations, but the populations did not significantly (*p* = 0.7723) differ in development time. Within SSA1-SG1, development time for *B. tabaci* colonies established from Kisubi, Kyabakadde, Nakisunga and Namulonge ranged from 28.1 ± 2.6 to 28.7 ± 2.3. *B. tabaci* colonies established from Kayingo had a slightly longer development time (31.6 ± 2.3) compared to the other four locations (Table 3). Within SSA1-SG2, *B. tabaci* colonies established from Kisubi (24.9 ± 2.6) and Kyabakadde (25.8 ± 2.3) had the shortest development time, followed by Kayingo (27.0 ± 2.3) and Nakisunga (28.4 ± 2.3). *B. tabaci* colonies established from Namulonge (41.1 ± 2.3 b) had a significantly (*p* < 0.05) longer development time compared to rest. The location × *B. tabaci* population interaction effect on development time was significant (*p* = 0.003).

## 4. Discussion

Field data correlates highly abundant *B. tabaci* whitefly populations infesting cassava in East and Central Africa with the presence of SSA1-SG1 [4,18,22,23], and recently, but to a lesser extent, with SSA1-SG2 whiteflies [19]. Since multiple factors affect whitefly population dynamics in the field, our study carried out comparative assays between four whitefly genotypes (SSA1-SG1, SSA1-SG2, SSA1-SG3 and SSA2) set up from single females in the laboratory to investigate whether the observed variations in field abundance are driven by biological traits for each genotype. There were no significant differences in the number of first instar nymphs, number of emerged adults, proportion of emerged female adults and the development time for SSA1-SG1 and SSA1-SG2 populations. Hence, we reject the hypothesis of the study, that SSA1-SG1 and SSA1-SG2 field abundances are driven by biological traits. Data suggest that the high field abundance of SSA1-SG1 and the low field abundance of SSA1-SG2 cassava whiteflies are not linked to the biological traits measured in this study, as SSA1-SG2 performed slightly better than SSA1-SG1 under controlled conditions in the insectary. The high abundance of SSA1-SG1 *B. tabaci* whiteflies in the field seems to be linked to other factors, such as a larger plant host range for this genotype compared to SSA1-SG2 [22].

This study has demonstrated that SSA1-SG1 and SG2 *B. tabaci* whiteflies possess significant (*p* = 0.0113) and non-significant differences (*p* = 0.0504) respectively, in the number of first instar nymphs compared to SSA1-SG3 *B. tabaci* whiteflies. Further, SSA1-SG1 and SG2 possessed a higher number of emerged adults than SSA1-SG3. Mugerwa et al. [34] study demonstrated that a SSA1-SG1 population collected from the Lake Victoria Basin of Tanzania possessed a significantly higher fecundity (number of eggs) than SSA1-SG3 population collected from the coastal region of Tanzania. In the Mugerwa et al. [33] study, the number of first instar nymphs was not measured, hence a direct comparison between the two studies cannot be made. Nevertheless, both the Mugerwa et al. [33] study and this study show that SSA1-SG1 possess a higher number of emerged adults than SSA1-SG3. SSA1-SG3 is the prevalent population in central and southern Tanzania as well as the coastal areas of East Africa, all regions where low whitefly populations are reported to occur generally [4,34,44]. Furthermore, field data obtained by Mugerwa et al. [22] showed that SSA1-SG1 (15.7%) was more prevalent than SSA1-SG3 (3.3%) among 870 whitefly adults collected from over 60 plant species in Uganda. No SSA1-SG3 whitefly was collected in the 98 whiteflies collected from cassava in the Mugerwa et al. [22] study in Uganda.

In addition to the reports of SSA1-SG3 species being less abundant in the field and less fecund under standardized conditions [25,34,45,46], parasitism could be another ‘biological brake’ contributing to the low field populations of this *B. tabaci* whitefly species [23,47,48]. Caution should be taken, however, in drawing conclusions from data obtained using a single SSA1-SG3 whitefly population. There remains a possibility that these data might not be a true representation of SSA1-SG3 populations generally. Examining our data further, we observed divergent results from one SSA1-SG2 population collected from Namulonge, which had a significantly longer development time than SSA1-SG2 populations collected from the other four locations.

SSA2 *B. tabaci* whitefly possessed similar biological traits with SSA1-SG1 *B. tabaci* whitefly in the insectary, which corroborates field reports of high whitefly abundance associated with the two species in East Africa [4,19,22,23]. A few studies reported SSA2 whiteflies as the highly abundant whitefly species on cassava in Uganda (between 1997 to 1999) [11], in some areas of northern Uganda [22,23] and in Juba, South Sudan [49]. The study indicated SSA2 *B. tabaci* whitefly to have a slightly higher proportion of emerged female adults. Production and maintenance of a higher female proportion in total progeny were among the reasons behind the displacement of *Rickettsia*-free Middle East-Asia Minor 1 (MEAM1) *B. tabaci* within a period of six years by *Rickettsia*-infected MEAM1 *B. tabaci* in the US [50]. However, currently, the SSA2 *B. tabaci* species has a lower occurrence in cassava fields than SSA1-SG1, which is not explainable by the data obtained in our study. The significant differences obtained for the SSA1-SG1 and SSA1-SG2 *B. tabaci* populations collected in five different locations in the Lake Victoria Crescent in Uganda is likely attributed to environmental factors. Other factors such as prevalent cassava varieties grown, host range and climate (not measured in this study) are probable drivers of the observed field whitefly abundances [23].

At a constant temperature of 28 ± 2 °C, 60% r.h. and 12:12 L:D, the average development time of 29.1 ± 1.2, 29.6 ± 1.2, 26.7 ± 3.7 and 32.2 ± 2.6 days were recorded for SSA1-SG1, SSA1-SG2, SSA1-SG3 and SSA2 *B. tabaci* populations respectively, in the current study. The results are similar to those by Legg [51], who reported a development time of 27–39 days for cassava whiteflies under field conditions in central Uganda, where annual average temperature and relative humidity are 25–31 °C and 66–80%, respectively. However, contrary to our finding, Aregbesola et al. [52] reported an average development time of 16.3 ± 0.6 for SSA1-SG3 whitefly in the laboratory using climatic chambers (Percival^®^ PGC-6L) set at a constant temperature of 28 °C, 65 ± 5% r.h. and 12:12 L:D. Furthermore, the authors reported an average development time of 21.3 days for the SSA1-SG3 *B. tabaci* population under field conditions in Dar es Salaam, Tanzania, where average temperature and relative humidity were 28 °C and 78%, respectively. The differences observed in the current study and that of Aregbesola et al. [52] could be attributed to several factors, including different crops and cassava varieties (Colombia vs. Albert), relative humidity conditions (60% vs. 65–78%) and duration of whitefly colonies in the laboratory used.

The shortest development time amongst all cassava whitefly populations was recorded on eggplant, taking ~25 days to complete their development on eggplant in contrast to ~35 days on cassava. Different development times are commonly observed for *B. tabaci* across different plant species [53,54]. For example, the development time of MEAM1 and Asia II-1 *B. tabaci* on five plant species (cotton, squash, tomato, tobacco and sweet potato) ranged from 21.0‒24.7 days and 20.6‒28.0 days, respectively [53]. The ability of the four whitefly populations to develop shorter on eggplant and presumably other crop and weed species highlights the importance of alternative hosts as sources for high whitefly populations that can invade cassava. While developing management practices to control whitefly populations and the viral disease they spread in cassava fields, it is of paramount importance to assess neighboring crop and weed host plants for their ability to serve as breeding sources for cassava whiteflies.

SSA1-SG2 *B. tabaci* colonies established from Namulonge had the longest development time (~41 days). SSA1-SG2 *B. tabaci* colonies from Kisubi, Kyabakadde, Nakisunga and Kayingo took ~25–28 days to complete their development. It is unclear why this SSA1-SG2 Namulonge colony had the longest development time compared to the other populations collected from Kisubi, Kyabakadde, Nakisunga and Kayingo. No differences were found in endosymbiont status that correlated with development time. PCR was used to screen for the primary endosymbiont *Portiera aleyrodidarum* and six secondary endosymbionts (*Arsenophonus*, *Cardinium*, *Fritschea, Hamiltonella*, *Rickettsia* and *Wolbachia*) in individual flies for each of the 12 colonies [55]. All flies tested positive for the primary endosymbiont but were negative for all the secondary endosymbionts except *Wolbachia*. The latter was found in all five SSA1-SG2 *B. tabaci* colonies and hence did not correlate with differences in development time. It should be stressed that high-throughput sequencing data has recently revealed that there are many more potential secondary endosymbionts in *Bemisia tabaci* [56], and hence, there may be a correlation with a new endosymbiont that was unknown at the time this study was undertaken. The long development time observed for the Namulonge *B. tabaci* colonies highlights the *mtCO1* marker not reflecting genetic differences that impact fitness. Unravelling the genetic factors behind the long development time of Namulonge *B. tabaci* colonies could possibly provide important information to assist the management of cassava whiteflies.

From the late 1980s, there has been a sharp increase in whitefly populations not only on cassava, but also on other crop and weed plant species in East Africa [1,4,11,19,45,46,57]. In the cassava agro-systems, the whitefly population surge has been attributed to several factors, including: (i) appearance of a highly fecund invasive whitefly [11], or the presence of distinct whitefly genotypes with high fecundity [34], (ii) virus–vector interactions on virus-infected cassava plants resulting in increased whitefly fecundity [12,13] and (iii) introduction of cassava disease-resistant and/or tolerant varieties which were highly susceptible to whitefly colonization [28]. This study supports previous ones that the population surges cannot be explained by whitefly genotype alone. The impact of climate change on the occurrence of whitefly populations has also been explored recently with a consistent relationship between *B. tabaci* abundance and suitable climatic conditions [58]; the increasing climate suitability in parts of East and Central Africa across the 39 years of the study shows a correlation not only with the increased prevalence of *B. tabaci* but also the devastating viruses transmitted by these whiteflies [58]. Of great concern presently is that West Africa, the major cassava-producing region in sub-Saharan Africa [59], has not yet witnessed superabundant populations despite this region having similar cassava mosaic begomoviruses and whitefly populations to those reported in East Africa [18,60,61,62]. Unravelling the factors contributing to the high whitefly populations in East Africa is of paramount importance to enable the development of whitefly management practices to prevent similar increases in whitefly abundance occurring in West Africa.

## Figures and Tables

**Table 1:** *Bemisia tabaci* colonies collected from cassava in Uganda and Tanzania and maintained on eggplant at the Natural Resources Institute, UK.

*Bemisia tabaci* Species	Country	Location	Agro-Ecological Zone	Coordinates	Collection Date
SSA1-SG1	Uganda	Kayingo	Lake Victoria Crescent	0.18828 N, 32.55647 E	February 2016
SSA1-SG1	Uganda	Kisubi	Lake Victoria Crescent	0.13449 N, 32.53751 E	February 2016
SSA1-SG1	Uganda	Kyabakadde	Lake Victoria Crescent	0.49756 N, 32.72995 E	February 2016
SSA1-SG1	Uganda	Namulonge	Lake Victoria Crescent	0.51716 N, 32.63612 E	February 2016
SSA1-SG1	Uganda	Nakisunga	Lake Victoria Crescent	0.34591 N, 32.76564 E	February 2016
SSA1-SG2	Uganda	Kayingo	Lake Victoria Crescent	0.18828 N, 32.55647 E	February 2016
SSA1-SG2	Uganda	Kisubi	Lake Victoria Crescent	0.13449 N, 32.53751 E	February 2016
SSA1-SG2	Uganda	Kyabakadde	Lake Victoria Crescent	0.49756 N, 32.72995 E	February 2016
SSA1-SG2	Uganda	Namulonge	Lake Victoria Crescent	0.51716 N, 32.63612 E	February 2016
SSA1-SG2	Uganda	Nakisunga	Lake Victoria Crescent	0.34591 N, 32.76564 E	February 2016
SSA2	Uganda	Kiboga	Lake Victoria Crescent	0.84068 N, 31.88285 E	August 2013
SSA1-SG3	Tanzania	Dar es Salaam,	Coast Zone	6.60789 S, 39.08093 E	February 2013

SSA1 and SSA2 refer to sub-Saharan Africa 1 and sub-Saharan Africa 2, while SG1, SG2 and SG3 refer to subgroup 1, subgroup 2 and subgroup 3 respectively, as defined by Legg et al. [4].

**Table 2:** Effect of host plant (eggplant and cassava) and *B. tabaci* populations (SSA1-SG1, SSA1-SG2, SSA1-SG3 and SSA2) on mean number of first instars, emerged adults, proportion of females in emerged adults and development time in the insectary (Natural Resources Institute, Gillingham, UK).

(a) Host Plant	No. of First Instars	No. of Emerged Adults	Proportion of Females in Emerged Adults	Development Time in Days
Cassava (N = 50)	58.8 ± 3.2 a	41.2 ± 2.9 a	0.44 ± 0.04 a	34.6 ± 1.0 b
Eggplant (N = 57)	53.0 ± 2.8 a	49.6 ± 3.3 a	0.41 ± 0.03 a	25.1 ± 0.9 a
**(b) *B. tabaci* Population**				
SSA1-SG1 (N = 44)	55.1 ± 3.2 ab	44.6 ± 3.3 ab	0.39 ± 0.04 a	29.2 ± 1.2 a
SSA1-SG2 (N = 48)	60.6 ± 3.4 b	50.9 ± 3.6 a	0.42 ± 0.03 a	29.6 ± 1.2 a
SSA1-SG3 (N = 5)	34.2 ± 6.1 a	32.0 ± 7.1 ab	0.51 ± 0.13 a	26.7 ± 3.7 a
SSA2 (N = 10)	45.8 ± 5.7 ab	32.6 ± 5.1 b	0.54 ± 0.09 a	32.2 ± 2.6 a

‘N’ is the number of replicates in a given treatment. Means followed by the same letters are not significantly different, while means followed by different letters are significantly different as separated by Tukey’s HSD test at *p* < 0.05.

**Table 3:** Development differences (number of first instars, emerged adults, proportion of females in emerged adults and development time) among SSA1-SG1 and SSA1-SG2 genotypes collected from five different locations (Kayingo, Kisubi, Kyabakadde, Nakisunga and Namulonge) in the insectary (Natural Resources Institute, UK).

Location	No. of First Instars		No. of Emerged Adults		Proportion of Females in Emerged Adults		Development Time in Days	
	SSA1-SG1	SSA1-SG2	SSA1-SG1	SSA1-SG2	SSA1-SG1	SSA1-SG2	SSA1-SG1	SSA1-SG2
Kayingo	43.7 ± 5.0 ab	69.9 ± 7.8 b	39.7 ± 5.4 ab	63.6 ± 8.4 b	0.68 ± 0.07 c	0.50 ± 0.06 ac	31.6 ± 2.3 ab	27.0 ± 2.3 a
Kisubi	65.3 ± 7.7 ab	68.0 ± 8.5 b	51.7 ± 7.3 b	57.8 ± 8.6 b	0.16 ± 0.05 b	0.38 ± 0.07 bc	28.6 ± 2.4 a	24.9 ± 2.6 a
Kyabakadde	47.6 ± 6.5 ab	54.9 ± 6.2 ab	32.7 ± 5.4 ab	48.5 ± 6.5 ab	0.16 ± 0.07 ab	0.35 ± 0.07 bc	28.6 ± 2.7 a	25.8 ± 2.3 a
Nakisunga	56.9 ± 6.4 ab	72.4 ± 8.0 b	44.5 ± 6.0 ab	58.9 ± 7.8 b	0.64 ± 0.07 c	0.43 ± 0.06 bc	28.7 ± 2.3 a	28.4 ± 2.3 a
Namulonge	62.1 ± 7.8 ab	39.1 ± 4.5 a	53.2 ± 7.9 b	26.9 ± 3.8 a	0.27 ± 0.07 ab	0.43 ± 0.09 bc	28.1 ± 2.6 a	41.1 ± 2.3 b

Number of replicates (N) for SSA1-SG1 and SSA1-SG2 was 10, except in SSA1-SG1-Kisubi, were N = 9, SSA1-SG2-Kisubi and SSA1-SG1-Namulonge were N = 8, and SSA1-SG1-Kyabakadde were N = 7. Means followed by the same letters are not significantly different, while means followed by different letters are significantly different as separated by Tukey’s HSD test at *p* < 0.05.

## Data Availability

Partial *mtCO1* sequences of the *B. tabaci* whiteflies obtained from the 12 established colonies used in this study were deposited in GenBank under accession numbers MW683071–MW683103. Raw data S1 and S2, and Scripts S1 and S2 are also attached.

## Supplementary Materials

The following are available online at https://www.mdpi.com/2075-4450/12/3/260/s1, Raw data S1: SSA1-SG1 and SSA1-SG2 development data. Raw data S2: SSA1-SG1, SSA1-SG2, SSA1-SG3 and SSA2 development data. Script S1: SSA1-SG1 and SSA1-SG2 R codes for analysis. Script S2: SA1-SG1, SSA1-SG2, SSA1-SG3 and SSA2 R codes for analysis.
